# Health systems as human systems: reflexivity, relationships, and resilience in the pursuit of the SDGs

**DOI:** 10.3389/fpubh.2025.1653839

**Published:** 2025-08-20

**Authors:** Lucy Gilson

**Affiliations:** ^1^Health Policy and Systems Division, School of Public Health, University of Cape Town, Cape Town, South Africa; ^2^Department of Global Health and Development, London School of Hygiene & Tropical Medicine, London, United Kingdom

**Keywords:** Health Policy and Systems Research (HPSR), health system strengthening, complex adaptive systems, health equity, leadership, reflexivity, resilience, Sustainable Development Goals (SDGs)

## Abstract

Health Policy and Systems Research (HPSR) plays a critical role in efforts to strengthen health systems in pursuit of the Sustainable Development Goals (SDGs). This manuscript, adapted from the 2024 Virchow Lecture, explores the nature of HPSR, presents a systems-thinking perspective on health systems, and outlines key principles and strategies toward health system strengthening. It emphasizes the human dimensions of health systems—relationships, trust, leadership, values and meaning-making—as foundational to their resilience and outcomes. This narrative is informed by decades of experience and research at the intersection of policy, practice, and academia, particularly in low- and middle-income countries. The paper concludes with a call to reimagine health systems as open, dynamic, and human-centered institutions that generate public value and promote equity.

## Introduction

In October 2024, I was honored to receive the Virchow Prize for Global Health alongside Prof. Johan Rockström. I see this award not primarily as a personal recognition, but rather as acknowledgment of the wider field of Health Policy and Systems Research (HPSR) and its diverse community of practitioners, researchers from various disciplines, frontline health workers, policymakers, managers, activists and advocates. Together we seek to “…*understand and improve how societies organise themselves in achieving collective health goals, and how different actors interact in the policy and implementation processes to contribute to policy outcomes”* in order to “*draw a comprehensive picture of how health systems respond and adapt to health policies, and how health policies can shape—and be shaped by—health systems and the broader determinants of health”* (1).

HPSR considers the health system as a whole, rather than focusing on specific health programmes or interventions. It also addresses activities beyond health care—both the inter-sectoral actions and the public policy interventions, such as alcohol and tobacco regulation, needed to address the social and commercial determinants driving many health challenges. The health systems lens, moreover, pays specific attention to the functions that underpin service delivery, collaboration and health policy implementation, such as governance, human resource development and management and financing. This breadth of focus has led the field to recognize health systems as complex adaptive systems—comprised of multiple interacting elements that together offer more than the simple sum of the parts (2). The focus on achieving *collective* health goals is, finally, a reminder that HPSR situates the health system as a societal platform for collective action toward social justice.

This manuscript is adapted from my Virchow Lecture and offers a personal yet grounded perspective on HPSR. I reflect on my professional journey and draw on empirical insights to consider what it means to view health systems as complex, adaptive, and fundamentally human. I also consider the implications of this perspective for health system strengthening and the pursuit of the SDGs.

## A personal and professional perspective

As a researcher and educator, I approach HPSR reflexively. I recognize the privileges conferred by my background, training, and the opportunity to traverse professional and disciplinary boundaries. I have lived and worked across geographies and organizations, and I draw from diverse fields including development studies, public policy, political science, public administration and health economics. I have also specifically sought to *learn with* those working in health systems and with households in communities. Their tacit knowledge is as critical as formal research evidence in seeking to strengthen these systems to respond to inter-locking health and wellbeing challenges. Indeed, “boundary spanning,” and an openness to different ways of seeing the world, are themselves distinguishing features of the HPSR field (3).

**Figure 1:**
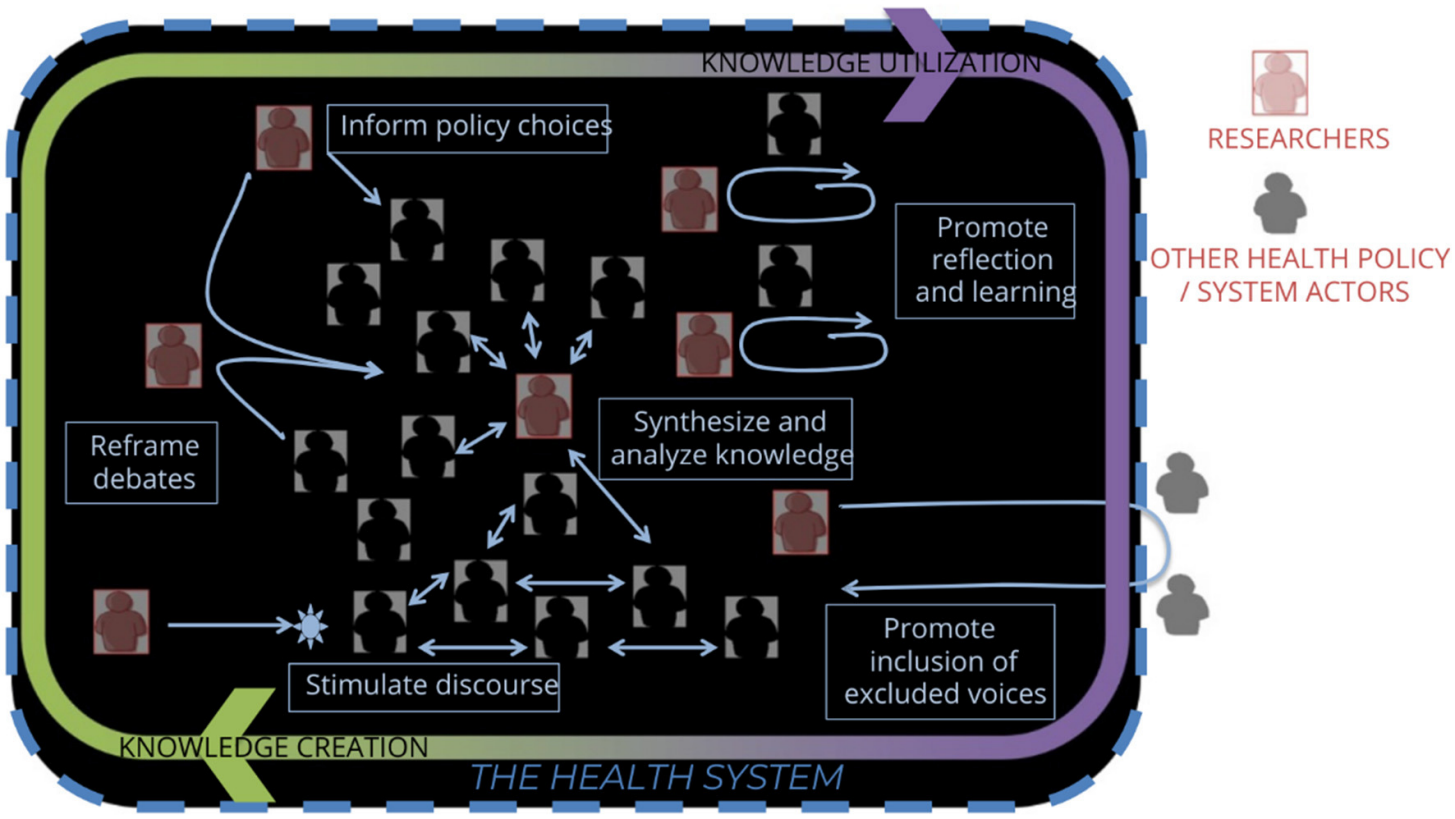
Health policy and systems research: a dialogic practice. Sheikh et al. (3).

In addition, as Figure 1 illuminates, researchers are one among many knowledge workers within health systems and in HPSR play roles that move beyond the traditional conception of a researcher.

My research, conducted mostly within national settings, has considered questions such as:

- What factors influence which health care providers people use, and how do the costs of accessing care impact on those who are most vulnerable?
- How do trust relationships impact on health worker motivation, and health system accountability?
- What organizational and political factors influence the implementation of policies, and encourage the emergence of innovation?
- How can systems leadership to support policy implementation be strengthened?

## Health systems as complex adaptive systems

A systems lens highlights five key features that characterize health systems as complex, adaptive (human) systems.

i. Actor-centric: Systems are composed of people and relationships.

Health systems exist to serve people and populations, including by supporting them in maintaining their own health. Experience also demonstrates that committed and motivated staff are the central resource of every health system—delivering health care and supporting engagement with other government and social actors (4). They make the many strategic and everyday decisions required to ensure that all the other resources needed to support these activities are available today and into the future. Delivering all health activities is also always about teamwork. Within and across organizations, people need to work together toward collective health goals. In essence, health systems operate through chains of relationships among people and teams (5).

ii. Relational: Trust and power dynamics shape system functioning.

The quality of human relationships is a key driver of health systems (5), strongly influenced by trust and power dynamics—which have consequences for the extent and outcomes of collective action (6, 7). For example, where health care teams are cohesive and relationships are respectful then patients are more likely to experience compassionate and effective care (4). Similarly, trusting relationships support the collaboration across organizational boundaries needed for inter-sectoral action to promote health and wellbeing. In contrast, where punitive managerial practices dominate and blame is prevalent, or where managers use their power to protect their own organizational territory, then relationships break down and activities falter (4). Patients and populations suffer the consequences. Societal power dynamics, moreover, drive the health inequities which health systems address.

iii. The everyday routines: Everyday values, norms and practices influence how people work together to generate system outcomes

Professional ethics clearly inform decision-making in health services, demonstrating how values drive everyday behavior. Nonetheless, the everyday practice of clinical and managerial leadership, bolstered by hierarchical norms, can undermine relationships and teamwork (8). For many in health systems, management meetings are another, often unseen, everyday practice. Although broadly intended to bring people together to enable collective action, meetings are often experienced as unproductive. For example, poorly organized and run meetings limit participants' opportunities to share experience and learn from each other, or to develop shared understandings of problems and how to address them (9). The opportunity to strengthen activities is then lost, and staff motivation can be undermined.

How you are treated in any relationship also signals meaning in terms of how you are valued by others; and how those with *more* power treat those with *less* power adds weight to the signal. Patients are often treated as passive recipients of medical expertise, rather than agents of their own health. Staff members who are repeatedly dis-respected by their manager may feel their sense of self-worth undermined—and then take out their frustration on others, even patients. In the reverse situation, however, being treated respectfully can build personal agency and confidence, helping to nurture the trusting provider-patient relationship that, in turn, supports patient care (6, 10).

iv. Meaning-making: People interpret their environments in ways that may reinforce or challenge everyday routines; these feedback loops either maintain the system as it is or catalyze possibilities of system change.

Differences in how people understand the same problem or possible responses to it, influence how well they work together to address the problem. In health care, important meaning-making often happens in the relationships between patient and provider, and between health worker and manager (including in meetings). It is in these relationships that shared understandings can be developed, or frustrated, with consequences for improved patient care or for health workers' commitment to change and to new policies (6, 10). What has been called the policy-implementation gap is often underpinned by communication weaknesses, and by the failure of those developing policies to understand the specific conditions in which policies are implemented (8).

v. Open systems: Health systems are influenced by their histories and external political, social, and economic forces.

The societal norms that shape health systems include those that reflect wider societal power dynamics and influence everyday routines and meaning-making, such as gender norms (11). Similarly, historical, socio-political and economic influences are reflected in current health system experience—and influence the trajectories of future health system reform (12, 13).

Together, these five features offer insights that dig beneath the surface of health systems as commonly discussed in global health. For the most part, the tendency is to focus on what has been called the health system building blocks (14)—such as levels of funding, physical infrastructure, numbers of staff, and availability of medicines. Whereas these building blocks emphasize what is sometimes called the system “hardware,” the features highlighted above consider the system “software” (15). System hardware and software interact and together influence system functioning. However, although important, the more intangible system software features are often overlooked and ignored in health policy debates and prescriptions.

## Health systems as value generators

**Figure 2:**
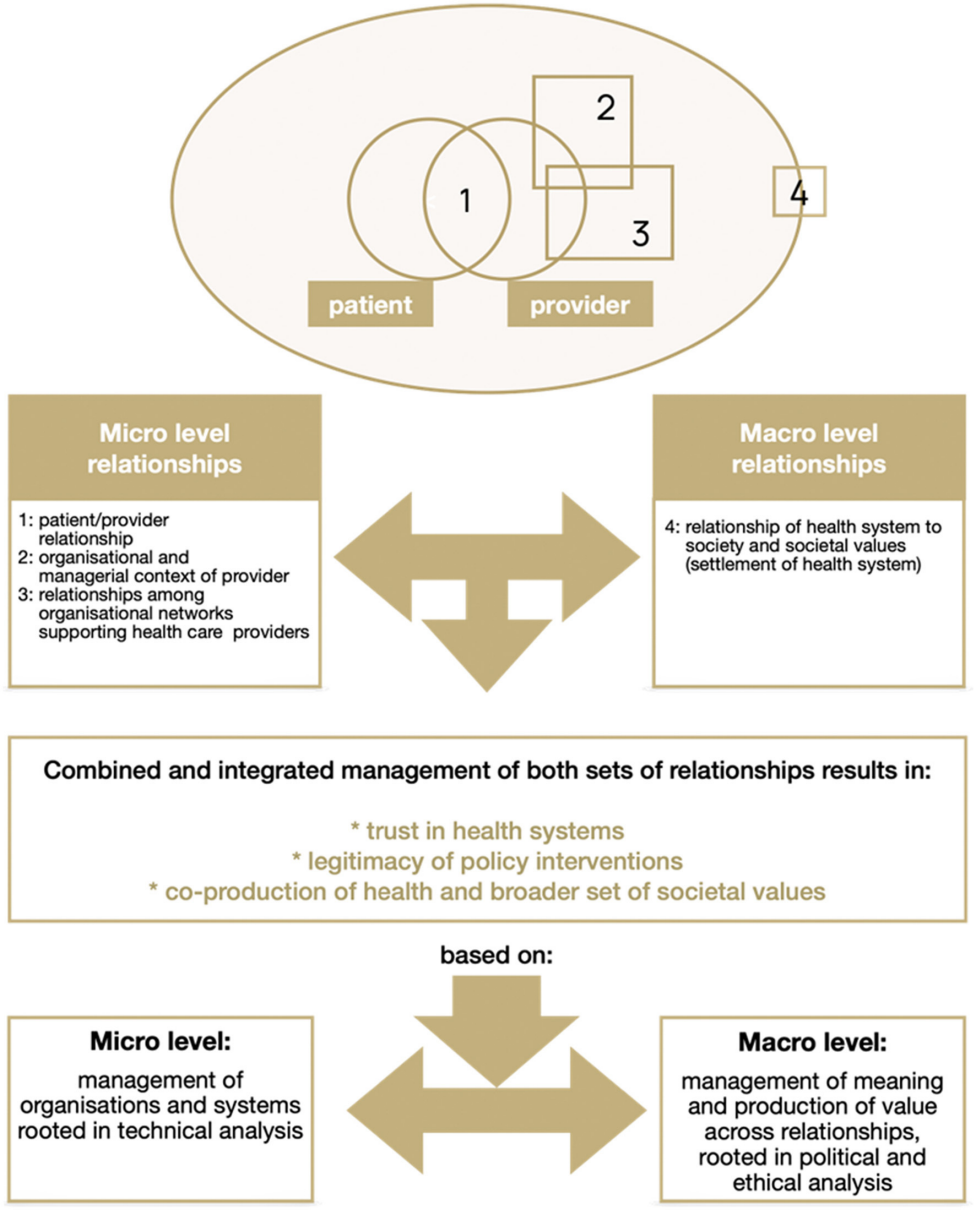
Managing health system relationships to build trust based on Gilson (6).

In reflecting on the role of trust in health systems, Figure 2 suggests not only that trust matters at the inter-personal and team (micro) level, but also at the macro or societal level. As part of the social fabric in every setting, experience in and of health systems impacts on social value. In the way they are organized and how they function every day, health systems may contribute to societal wellbeing. They may support social solidarity, economic development and environmental sustainability, for example (16). Experience also demonstrates that health systems can generate political trust (17). In addition, inclusive and ethical practices—which support trusting relationships—have the potential both to address the health effects of discrimination (18) and foster public value (19). This potential is why HPSR understands health systems as a societal platform for collective action toward social justice.

However, infused with power dynamics and societal norms, health systems often work to perpetuate exclusion and inequity (20).

Research on user fees, for example, has revealed how having to pay out of pocket payments for healthcare is impoverishing—not only in an economic sense but also as it undermines the capability to claim essential services. The experience signals the exclusion of more economically vulnerable groups and undermines their trust in the health system (21). Conflict within health system relationships can also be exclusionary because, as noted, how people are treated signals value. In maternity care, for example, disrespect and abuse has been widely recognized as a violation of women's dignity, risking health harms, driven by the ways wider socioeconomic inequality is embedded in institutional structures and processes (22).

Further, exclusion is signaled through the way decisions are made (6). What is termed “control and command” decision-making is still the usual practice in many health systems, marginalizing community organizations and patients (23), as well as frontline health workers (8), in decision-making. The weak engagement of communities, especially the most vulnerable groups, in developing activities intended to improve their health not only limits the potential for these activities to be effective, but also sustains existing power disparities (18).

## Toward health system strengthening

The challenges that health systems seek to address, from non-communicable diseases to mental health, gender-based violence, or the impacts of climate change, are themselves multifactorial and demand complex responses. At the same time, the features that make health systems dynamic and potentially responsive to these challenges, mean they are difficult to steer through the top-down decision-making and reform that is common across countries (24). Yet global health policy discourse tends to view health systems both as focused only on illness and treatment, rather than prevention, and as machines to be fixed through centrally- or externally led, and technocratic, reforms, often focused on health system hardware (24, 25).

New strategies for health system strengthening are needed as we consider the SDGs. These strategies must support the development of the systemic resilience required to adjust and adapt to constant pressure and changing needs, and still continue to deliver health, wellbeing and wider public value. Developing such resilience requires adequate resourcing. Also important, however, are: investment in relationships; the changes in everyday routines that enable trust-building and support shared understandings to be developed across the chains of people and teams that work together within the health system; and recognition of health systems as open systems (26, 27).

Three priority areas for health system strengthening are, therefore:

i. Inclusive Decision-Making: Health system equity and resilience is supported through inclusive and ethical decision-making. Although challenging to initiate and sustain, participatory governance mechanisms can channel community voice, whilst active community and health worker groups must claim decision-making space. Experience during COVID-19 (23), for example, demonstrated the value and gains of community-led responses to health challenges. Appropriately disaggregated data are also needed to support decision-making (5, 20).
ii. Leadership at the Frontline: The local level is the frontline of response to all current and emerging health threats (28, 29). Responding to threats requires collaboration among community, social and government actors, and this is enabled by the forms of leadership that sustain trusting relationships and nurture collective power and innovation. This leadership must, further, be supported by purposeful system-level capacity development activities (30), including the governance structures and resourcing that enable local actors to work together in the interests of their communities (28).
iii. Global Action for Social Justice: As open systems, health systems are impacted by global and structural inequities. These drive the commercial forces influencing the marketisation of health care and growing burden of non-communicable diseases and were reflected in the vaccine inequity experienced during the COVID-19 pandemic. They demand coordinated international responses and global networks of solidarity (31, 32).

Taking action in all three areas will inevitably be challenging. It will confront the power dynamics and societal norms embedded in health systems that, as already noted, work to perpetuate exclusion and inequity. Yet these areas of action are critical leverage points for system change, and there are positive experiences from which to learn, and on which to build, as highlighted in the experiences cited.

## The role of researchers and educators

Researchers, as system actors, are embedded in the systems we seek to study and support (see Figure 1). We are accountable not only for the knowledge we generate but for how we engage with others in its generation. As our choices carry ethical and political weight we must exercise those choices with care. We must consider not only what issues to address in research but also with whom we carry out research and how we carry it out (33). Inclusive and participatory research processes are, for example, themselves important in supporting health system strengthening (34).

## Conclusion

Health systems are deeply human. They are constituted by relationships, values, and routine practices. To strengthen them does not simply entail implementing technical solutions but instead requires that attention be paid to enabling relationships that foster trust, inclusion, and public value. The African principle of Ubuntu reminds us: a person is a person through persons.

In this light, the work of HPSR is political, relational, and profoundly hopeful. It calls us to nurture systems capable of delivering on the promise of the SDGs, not just through what they do, but how they do it.

## Data Availability

The original contributions presented in the study are included in the article, further inquiries can be directed to the corresponding author.
